# Quantification of left ventricular ejection fraction using through-time radial GRAPPA for real-time imaging

**DOI:** 10.1186/1532-429X-15-S1-P45

**Published:** 2013-01-30

**Authors:** Vidya Nadig, Victoria Yeh, Vikas Gulani, Robert C Gilkeson, Nicole Seiberlich

**Affiliations:** 1 Cardiology, Metrohealth Campus of Case Western University, Cleveland, OH, USA; 2 Case Western University School of Medicine, Cleveland, OH, USA; 3 Radiology, University Hospitals of Cleveland, Cleveland, OH, USA; 4 Biomedical Engineering, Case Western University School of Medicine, Cleveland, OH, USA

## Background

Cardiac MRI requires a steady cardiac rhythm for ECG-gating, and multiple breath-holds to minimize respiratory artifacts. By employing a non-Cartesian parallel imaging in form of through-time radial GRAPPA [1], accelerated imaging can be performed with temporal resolutions of < 50 ms, obviating the need for gating or breath-holding. breath-holding. We tested the hypothesis that volumetric measurements in the LV obtained with the real-time method could replace traditional measurements.

## Methods

A total of 31 subjects (23 patients, 8 volunteers) were scanned on a 1.5T Avanto or 1.5T Espree scanner (Siemens Medical Solutions, Erlangen, Germany) using a combined spine and abdominal receiver array with 12 to 15 channels. The gold-standard cardiac functional examination was performed in a short-axis orientation with ECG gating, requiring 12-16 breathholds with the following parameters: Cartesian bSSFP sequence, TR~31-62 ms, in plane resolution = 1.4-2.6 mm^2^, slice thickness = 6-8 mm, cardiac phases = 18-30. The real-time scans (26 calibration frames per slice and accelerated acquisition) were performed immediately following the gold-standard scan with no ECG gating or breath-holding: radial bSSFP sequence, TR= 2.64 ms, resolution = 2.3 mm^2^, slice thickness = 6-8 mm. A data acceleration factor of R=8 (16 projections for 128 x 128 matrix) was used such that the temporal resolution for real-time imaging was 42.2 ms per image. Calibration data for non-Cartesian GRAPPA reconstruction was acquired without ECG gating or breath-holds. After data collection and reconstruction, the blood volume in the left ventricle was assessed to determine the ESV, EDV, and EF for both methods.

## Results

Bland-Altman analysis [2] was used to analyze the agreement between the two methods. This showed that 30 of the 31 of the EF measurements using traditional breath-hold imaging and the real-time free breathing method were within the 95% limits of agreement. The mean difference in LVEF between the two methods was -2% (breath-hold minus real-time). All 31 measurements of EDV using both methods were within 95% limits of agreement. The mean difference in EDV between the two methods was -4ml. 30/31 measurements of ESV using both methods were within the 95% limits of agreement. The mean difference in ESV between the two methods was 2 ml. The differences in EF, EDV, and ESV are not clinically significant [3].

## Conclusions

Our results show no significant statistical or clinical difference between volumetric analysis determined using standard breathhold cine imaging and through-time radial GRAPPA. This indicates that standard method can be replaced by the real-time imaging approach which can be used even for patients with arrhythmia or difficulty with breath-holding.

## Funding

This work was funded by Case Western Reserve University/Cleveland Clinic CTSA UL1 RR024989, and NIH/NIBIB R00EB011527.

**Figure 1:**
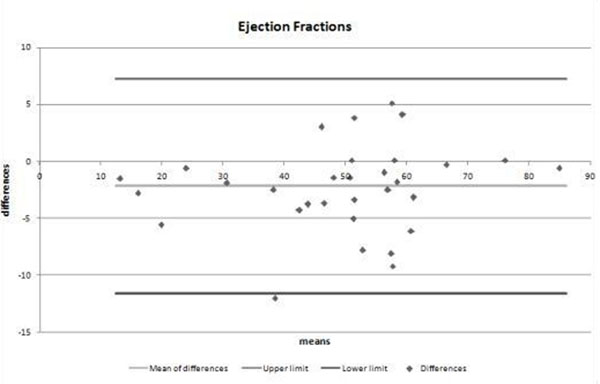
Bland Altman plot of agreement of ejection fractions measured by standard breath-hold technique versus real-time free-breathing technique.

**Figure 2:**
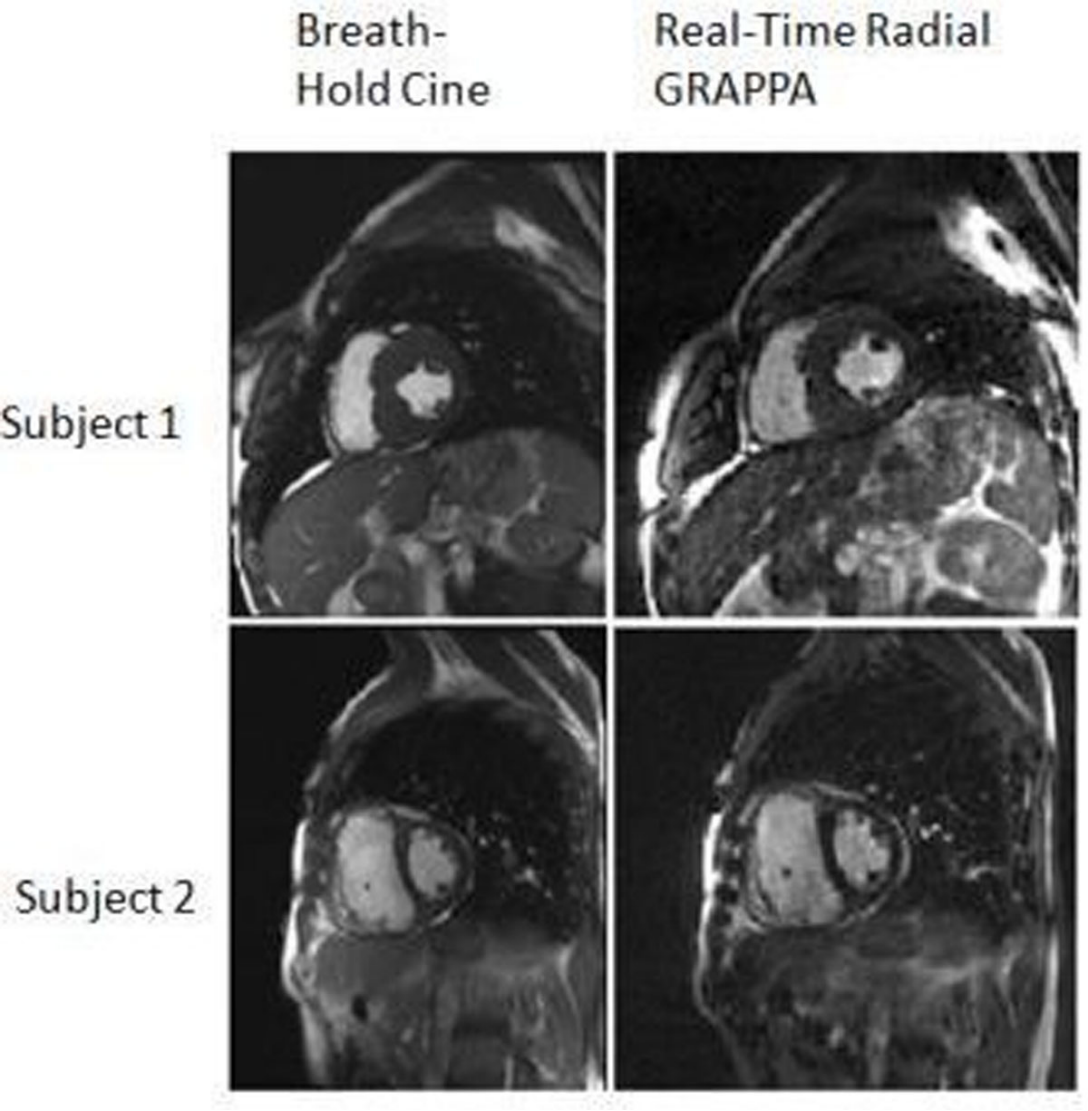
Short axis images of the left ventricle obtained by standard breath-hold method and real-time radial GRAPPA.
